# Usability Evaluation of Urinary HAI-1, STMN-1 and TN-C in the Diagnosis of Bladder Cancer

**DOI:** 10.3390/jcm14113664

**Published:** 2025-05-23

**Authors:** Beata Szymańska, Bartosz Małkiewicz, Janusz Dembowski, Agnieszka Piwowar

**Affiliations:** 1 Department of Toxicology, Faculty of Pharmacy, Wroclaw Medical University, 211 Borowska Street, 50-556 Wroclaw, Poland; agnieszka.piwowar@umw.edu.pl; 2 Department of Minimally Invasive and Robotic Urology, Centre of Excellence in Urology, Wroclaw Medical University, 213 Borowska Street, 50-556 Wroclaw, Poland; bartosz.malkiewicz@umw.edu.pl (B.M.); janusz.dembowski@umw.edu.pl (J.D.)

**Keywords:** bladder cancer, hepatocyte growth factor activator inhibitor type 1, stathmin 1, tenascin C, diagnostic markers

## Abstract

**Background**: Proteins with different functions, such as Hepatocyte growth factor activator inhibitor type 1 (HAI-1), Stathmin 1 (STMN-1), and Tenascin C (TN-C), whose activity has been observed in various types of cancers, inspired our study in bladder cancer (BC) patients. The aim of the study was to evaluate selected parameters and their combinations in the diagnosis of BC. The study took into account the degree of invasiveness and malignancy of BC. Based on the analysis of the Receiver Operating Characteristic Curve (ROC), the diagnostic value of single parameters and their combinations as potential indicators of BC was assessed. **Patients and Methods**: The research material consisted of urine samples from patients with BC, and urine samples from a control group without urological diseases. The concentrations of the examined parameters were measured using an immunoenzymatic method. **Results**: Statistically significant higher concentrations of HAI-1 (*p* ≤ 0.001), STMN-1 (≤0.001) and TN-C (0.002) were found in the patients with BC compared to the control group. Strong relationships were shown between these parameters. ROC analyses showed that the best single parameter for detecting BC is STMN-1, and in the combination of HAI-1+STMN-1. The highest diagnostic value was obtained for the combination of HAI-1+STMN-1 in the patients with high malignancy (sensitivity 82%, specificity 91%). **Conclusions**: Preliminary studies of parameters have shown their utility as potential markers in BC, especially of STMN-1 and combinations HAI-1+STMN-1. However, to learn more about the contribution of these parameters to the progression of bladder cancer, it would be appropriate to continue the studies.

## 1. Introduction

Bladder cancer is one of the most common cancers of the urinary system in the elderly. It is four times more common among men—in 98% of cases after 45 years of age [1]. Risk factors predisposing to the occurrence of abnormalities in the cell genome, and consequently leading to the development of BC, are: male sex, older age, Caucasian race, chronic infection or irritation of the bladder, smoking, exposure to arsenic in drinking water, and occupational exposure to certain chemical compounds, such as aromatic amines, polycyclic aromatic hydrocarbons, nitrosamines, organochlorine derivatives and carbamates contained in pesticides and cadmium compounds [2,3]. An important role is also assigned to the inflammatory processes accompanying infections with Schistosoma haematobium, whose pathogen is a common cause of bladder cancer in the regions of Africa and the Middle East [4]. Antitumour therapy with cyclophosphamide should also be classified as an iatrogenic risk factor due to its toxic metabolite, acrolein, which strongly irritates the urothelial epithelium [5]. Radiation therapy within the pelvic organs is also not neutral [6]. Recently, increasing attention has been paid to the presence of factors that may be associated with the presence of cancer, including BC, such as obesity, hyperglycemia, hypertension and hypertriglyceridemia, which cause inflammation in the body. Another potential etiological factor is antihypertensive drugs, anticoagulants and statins, used alone or in combination, but their effect, especially on BC carcinogenesis, is still not fully understood. A positive association has been shown between cardiovascular disease (CVD) and the occurrence of BC. CVD was an independent protective factor for BCa. However, this effect has not been confirmed in the case of high-risk tumours [7].

BC is characterised by a lack of specific signals that indicate a cancer is taking place in the body. Currently, the diagnosis and assessment of the stage of bladder cancer are: an interview with the patient, physical examination, cystoscopy, imaging diagnostics (e.g., ultrasound of the abdominal cavity, computed tomography, urography, magnetic resonance imaging), cystoscopy and examination of the composition of the urine sediment [8]. Due to the imperfection and invasiveness of the above-mentioned methods of BC diagnosis, new ones are constantly being sought, which are distinguished by less invasiveness, high specificity and sensitivity, low costs and easy access to biological material. Despite many attempts to find a diagnostic indicator for detecting BC, no marker has been found that would significantly improve its diagnostics, therefore any research in this area seems justified [9,10,11,12].

Different in terms of protein functions, such as Hepatocyte growth factor activator inhibitor type 1 (HAI-1) [13,14,15,16], Stathmin 1 (STMN-1) [17,18,19,20] and Tenascin C (TN-C) [21,22,23,24,25], whose activity has been observed in various types of cancer, inspired us to study them in bladder cancer.

HAI-1, also known as Kunitz type 1 serine protease inhibitor, is expressed on the surface of epithelial cells. HAI-1, also known as the Kunitz serine protease inhibitor type 1, is a transmembrane serine protease inhibitor that is expressed on the surface of epithelial cells [26]. HAI-1 is essential for cell growth, survival, and mobility. It plays a significant regulatory role in the proteolytic reaction, which is essential in the formation of active proteins involved in the functioning of various cells. It plays a significant regulatory role in the proteolytic reaction, which is an important mechanism for the production of biologically active proteins mediating various cellular functions [27]. HAI-1 has been shown to inhibit a number of type II transmembrane serine proteases (TTSPs), including matriptase, hepsin, TMPRSS13 (transmembrane serine protease 13) and HGFA (hepatocyte growth factor activator), which are mainly pro-protease activating proteases of HGF (precursor of hepatocyte growth factor) [28]. It has been shown that increased activity of proteases in neoplastic tissue contributes to cancer progression [29]. Insufficient levels of HAI-1 may result in dysregulation of protease activity and thus lead to aggravation of the neoplastic process. Thus, HAI-1 exerts an inhibitory effect on cancer invasion, metastasis, and processes that result in poor prognosis in cancer patients [30].

STMN-1 belongs to the family of microtubule-regulating proteins, also known as oncoprotein 18 (Op18) is a cytosolic phosphoprotein [31]. Regulation of dynamic microtubule instability is critical to cellular processes, from mitosis to membrane transport. The mechanism of action of STMN-1 remains a contentious and controversial issue. Two models of the STMN-1 mechanism have been proposed for the function of the indexing spindle. One model says that STMN-1 reduces MT (microtubule) polymer by sequestering tubulin, indirectly promoting the ‘catastrophe’ that is the transition from growth to depolymerisation [32,33]. Another model assumes that STMN-1 causes the ‘catastrophe’ directly, possibly acting at the ends of the microtubules. The second mechanism claims that STMN-1 changes the dynamics of microtubules by directly destabilising growing microtubules [34,35]. STMN-1, in addition to its function of destabilising microtubules, may contribute to promoting the malignant potential of neoplastic cells by initiating the EMT (epithelial–mesenchymal transition), i.e., an epithelial–epithelial transition during which epithelial cells may acquire a mesenchymal-like phenotype. Under pathological conditions, EMT is observed during tissue fibrosis and in neoplasms [36].

TN-C is a glycoprotein of the extracellular matrix that has been assigned a diverse range of functions. TN-C plays an important role during morphogenesis in embryonic life, where its high expression decreases shortly after the baby is born. Another rapid increase in TN-C occurs during inflammatory processes and fibrosis, in which the repair function of this protein has been demonstrated. Elevated expression of TN-C is detected in pathological conditions such as chronic inflammation (e.g., rheumatoid arthritis), autoimmune diseases, and cancer. In the last two decades, evidence has emerged showing a significant role of TN-C in heart and arterial damage, tumour angiogenesis and metastasis, and in modulating the behaviour of stem cells [37,38,39,40].

In the presented study, the concentration of the tested parameters in the urine of patients with BC and in a group of urologically healthy people constituting a control group was carried out. The invasive and malignant stage of BC was taken into account. Interrelationships between these proteins were also investigated. Based on a ROC (Receiver Operating Characteristic Curve) analysis, the initial diagnostic value of HAI-1, STMN-1 and TN-C and their combinations as potential markers of BC were assessed.

In the available literature, individual information was found on the examined proteins—HAI-1, STMN-1 and TN-C—in bladder cancer. Until now, the interrelationships between these proteins have not been studied. A single parameter is often insufficient for a proper diagnostic assessment; therefore, the combination of three proteins that differ in terms of structure and function may prove to be a good choice in the search for a potential non-invasive (urine was used for the test) diagnostic panel in BC.

## 2. Materials and Methods

### 2.1. Patient Selection and Collection of Data

The study group consisted of BC patients in the Urology and Oncological Urology Department of Wroclaw Medical University and people without BC constituting the control group.

The inclusion criteria for the study group was confirmed presence of bladder cancer. The exclusion criteria for the study group were the presence of other cancers, diseases of the genitourinary system such as urinary stones, active bladder infection and prostate diseases. The inclusion criteria for the control group were no cancers including BC, no active bladder infections, no diseases of the genitourinary system such as urinary stones, or prostate diseases and age similar to the study group.

This study was conducted in accordance with the Declaration of Helsinki. The study was approved by the Ethics Committee of the Medical University of Wroclaw (no. KB-276/2016). In the Department of Pathomorphology and Oncological Cytology of the Medical University of Wroclaw, histopathological tests of tissues collected from patients were performed to determine the degree of invasiveness and malignancy of BC. The patients were divided into subgroups based on the stage of the tumour into low-grade (LG) and high-grade (HG) according to the WHO/International Society of Urological Pathology—ISUP System 2004 classification, and subgroups of patients with non-muscle-invasive bladder cancer (NMIBC) and muscle-invasive bladder cancer (MIBC) according to the TNM (tumour, nodules, metastases) classification developed by the Union for International Cancer Control (UICC) in 2009.

### 2.2. Material and Methods

The material for laboratory testing of the selected parameters (HAI-1, STMN-1, TN-C) consisted of urine from the BC patients and the control group. The urine samples were collected in polystyrene containers (Aptaca, Canelli, Italy), and then centrifuged by an MPW-350 laboratory centrifuge (MPW Instruments, Warsaw, Poland) for 10 min (1500× *g*). The obtained plasma was then aliquoted and placed in test tubes. The material was frozen at −80 °C and remained so preserved until the parameters were determined. The immunoenzymatic method was used to measure the concentration of the tested proteins in the urine of patients and control group with Enzyme-Linked Immunosorbent Assay Kits: Human hepatocyte growth activator inhibitor 1 (HAI-1), ELISA kit, Shanghai Coon Koon Biotech Co., Ltd. (Shanghai, China); Human Stathmin 1 (STMN-1), ELSA kit, Bioassay Technology Laboratory, Shanghai (Shanghai, China); Human Tenascin C (TN-C), ELISA kit, Shanghai Coon Koon Biotech Co., Ltd., according to the manufacturers’ instructions in the listed test. The HAI-1/STMN-1/TN-C was immobilised with a human-specific HAI-1/human/STMN-1/human/TN-C monoclonal antibody and was detected using a human-specific HAI-1/human/STMN-1/human/TN-C/human polyclonal antibody conjugated to horseradish peroxidase (HRP). In each assay, the reaction was developed using a substrate solution (chromogen A and B). The substrate reaction was stopped, and the absorbance was measured at 450 nm with the correction read at 540 nm using an ELISA reader. The kits provided standards ranging from 0 ng/mL to 800 pg/mL for HAI-1, 0 ng/mL to 30 ng/mL for STMN-1, and 0 pg/mL to 24 pg/mL for TN-C. To calculate the concentrations of the measured parameters, standard curves were used, plotted according to the point-to-point reading of the standard concentrations against the measured absorbances.

Concentrations of parameters in urine were calculated in relation to the urine creatinine concentration estimated by Jaffe’s routine method based on the reaction of picric acid (Picric Acid, Saint Louis, MO, USA, SIGMA, Cat. No. 319287).

Results were reported in ng/mg creatinine. The values of the concentration of the examined parameters in the urine were converted to the concentration of urinary creatinine in order to eliminate the influence of dilution or concentration.

### 2.3. Statistical Analysis

All statistical analyses were carried out using TIBCO Software Inc., Palo Alto, CA, USA (2017). Statistica (data analysis software system), version 13.3 with the additional Plus Package was also used. Lilliefors and Kolmogorov-Smirnov tests were used to check the distribution of the concentrations of the studied parameters. Since a nonparametric distribution of concentrations was obtained, the nonparametric Mann–Whitney U test was used to compare the values of variables between the study and control groups. The Kruskal-Wallis test was used to check the differences in the values of variables in the subgroups of patients (NMIBC, MIBC, LG and HG) and controls with post hoc analysis (Bonferroni test). Interdependencies between continuous variables were checked with the Spearman test. Receiver operating characteristic (ROC) curves were plotted and the area under the curves (AUC), cut-off points, sensitivity and specificity of the tests were calculated, which allowed for the assessment of the diagnostic value of the studied parameters. In all analyses, *p* < 0.05 was accepted as a significant value.

## 3. Results

### 3.1. Study Population

The study group consisted of 56 BC patients with a mean age of 69 years. The control group included 32 healthy volunteers with a mean age of 67 years. No statistically significant differences in characteristic features were observed between patients and the control group. The characteristics of the examined groups are shown in Table 1. The age and sex of the BC patients and the control group were not statistically different (*p* > 0.05).

The obtained values of the studied parameters in the urine of the BC patients and the control group were converted to values of creatinine determined in urine to take into account their dilution. In the BC patients, the mean concentration of creatinine in the urine was 1.18 ± 0.54 mg/mL and 1.55 ± 0.44 mg/mL in the control group, and the differences were not significantly different (*p* > 0.05).

### 3.2. Results of HAI-1, STMN-1 and TN-C in Urine of BC Patients and Control Group

**Figure 1:**
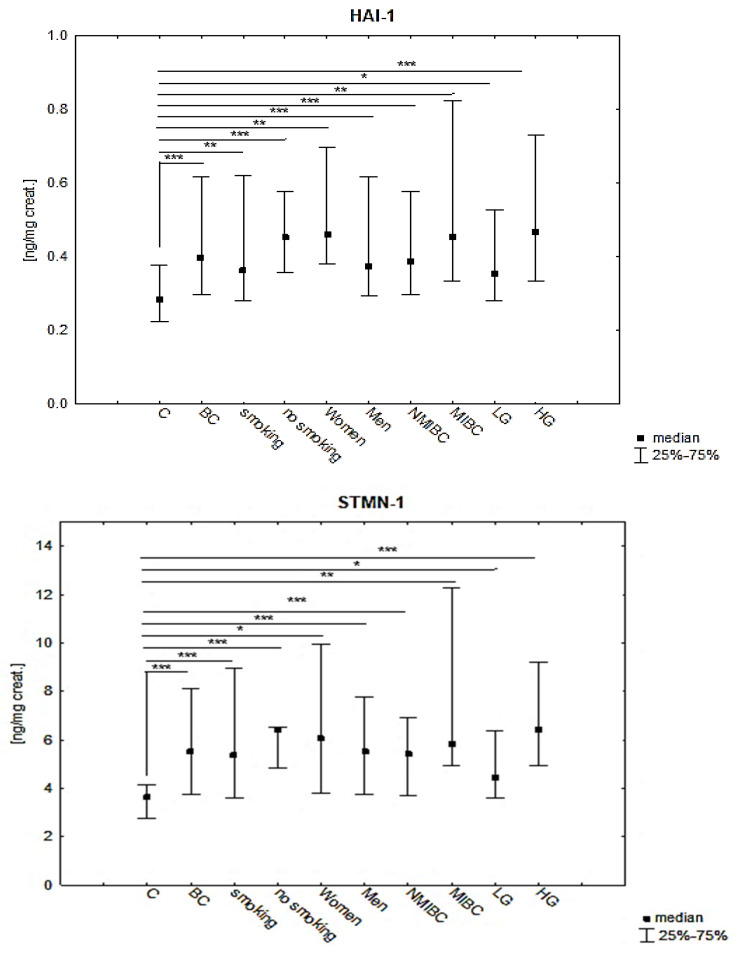

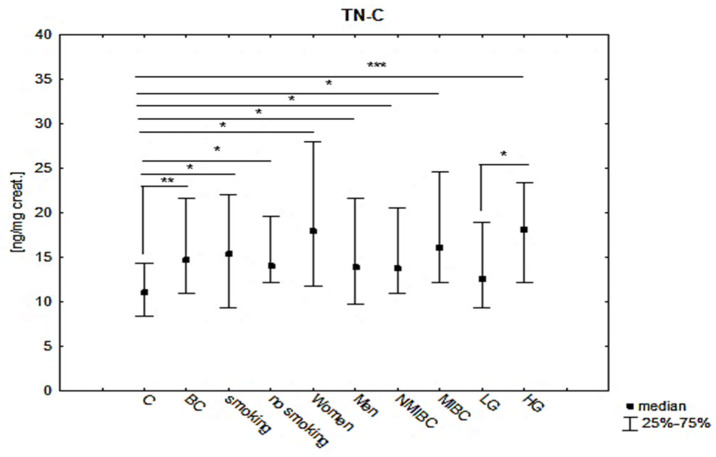
Distribution of HAI-1, STMN-1 and TN-C levels in BC patients and in BC subgroups, with statistically significant differences compared to control group (C); *** ≤0.001, ** ≤0.005, * ≤0.01. HAI-1—Human hepatocyte growth activator inhibitor 1; STMN-1—Stathmin 1; TN-C—Tenascin C; BC—bladder cancer; C—control group; NMIBC—non-muscle invasive bladder cancer; MIBC—muscle-invasive bladder cancer; LG—low grade; HG—high grade.

The mean values, standard deviations, medians with an interquartile range (IQR) for HAI-1 obtained in the urine of the BC patients, subgroups and control group, and the significance (*p*) values are presented in Table 2 and Figure 1.

There was a statistically significant difference between the values of the HAI-1 concentrations in the urine of the patients with BC (1.9-fold higher) compared to the control group (*p* ≤ 0.001). Analysing the HAI-1 concentrations in the subgroups of patients with different degrees of cancer invasiveness in relation to the control group, a statistically significant difference was shown, K-W: H (2, N = 88) = 15.75; *p* = 0.0004. However, no significant difference was found between the subgroups, although the mean concentration HAI-1 in MIBC was 1.2-fold higher than in NMIBC. Investigating the significance between the subgroups of patients with different cancer grades, statistically significant differences were found between the HAI-1 concentrations in subgroups compared to the control, K-W: H (2, N = 88) = 19.07; *p* = 0.0001, but no significance was found between the subgroups. In the HG subgroup, the HAI-1 concentration was 1.7-fold higher than in the LG subgroup. A higher concentration of HAI-1 was observed in the patients who smoked cigarettes compared to the non-smoking patients, but the difference was not statistically significant. In the women with BC, the concentration of HAI-1 was almost twice as high as in the men, but it was not statistically different.

The mean values, standard deviations, medians with an interquartile range (IQR) for STMN-1 obtained in the urine of the BC patients, subgroups and control group, and the significance (*p*) values are presented in Table 3 and Figure 1.

There was a statistically significant difference between the concentrations of STMN-1 in the patients with BC (2.1-fold higher) in relation to the control group (*p* ≤ 0.001). Analysing the concentrations of STMN-1 in the subgroups of patients with different degrees of cancer invasiveness, in relation to the control group, statistically significant differences were observed, K-W: H (2, N = 88) = 19.95; *p* = 0.0000. However, no significant difference was found between the subgroups, although the mean STMN-1 concentration in MIBC was 1.3-fold higher than in NMIBC. When examining the significance between the subgroups of patients with different degrees of cancer malignancy compared to the control group, significant differences were also shown, K-W: H (2, N = 88) = 24.17; *p* ≤ 0.001, however, no significance was found between the subgroups in relation to each other. In the HG subgroup, the mean STMN-1 concentration was 1.7-fold higher than the mean concentration in the LG subgroup, though not significantly. The STMN-1 levels were slightly different in the smoking patients and the non-smoking patients. In the women, the STMN-1 levels were 1.7-fold higher than in the men, but the difference was not statistically significant.

The mean values, standard deviations, medians with an interquartile range (IQR) for TN-C obtained in the urine of the BC patients, subgroups and control group, and the significance (*p*) values are presented in Table 4 and Figure 1.

There was a statistically significant difference between the TN-C concentrations in the urine of the patients with BC (1.8-fold higher) compared to the control group (*p* = 0.002). The concentrations of TN-C in the subgroups of patients with different degrees of cancer invasiveness differed significantly compared to the control group K-W: H (2, N = 88) = 10.57; *p* = 0.005 However, no significant difference was found between the subgroups, although the mean TN-C concentration in MIBC was 1.2-fold higher than in NMIBC. When examining the significance between the subgroups of patients with different degrees of cancer malignancy and the control group, a statistically significant difference was also shown, K-W: H (2, N = 88) = 15.92; *p* = 0.0003. In the LG subgroup, no statistically significant difference was found compared to the control, but there was a significant difference between the TN-C concentrations in the HG subgroup versus LG. In the HG subgroup, the mean TN-C concentration was statistically significantly higher (1.8-fold higher) than the mean concentration in the LG subgroup. The TN-C concentration was 1.3-fold higher in the smoking BC patients and 12.2-fold higher in the women, but these differences were not significant.

### 3.3. Correlations

Mutual positive correlations between HAI-1 STMN-1 and TN-C in the BC patients and subgroups are demonstrated in Table 5 (the correlations were evaluated with Spearman’s nonparametric test).

The conducted studies have shown strong positive correlations between the HAI-1, STMN-1 and TN-C parameters in the BC patients and their subgroups (NMIBC, MIBC, LG and HG).

**Figure 2:**
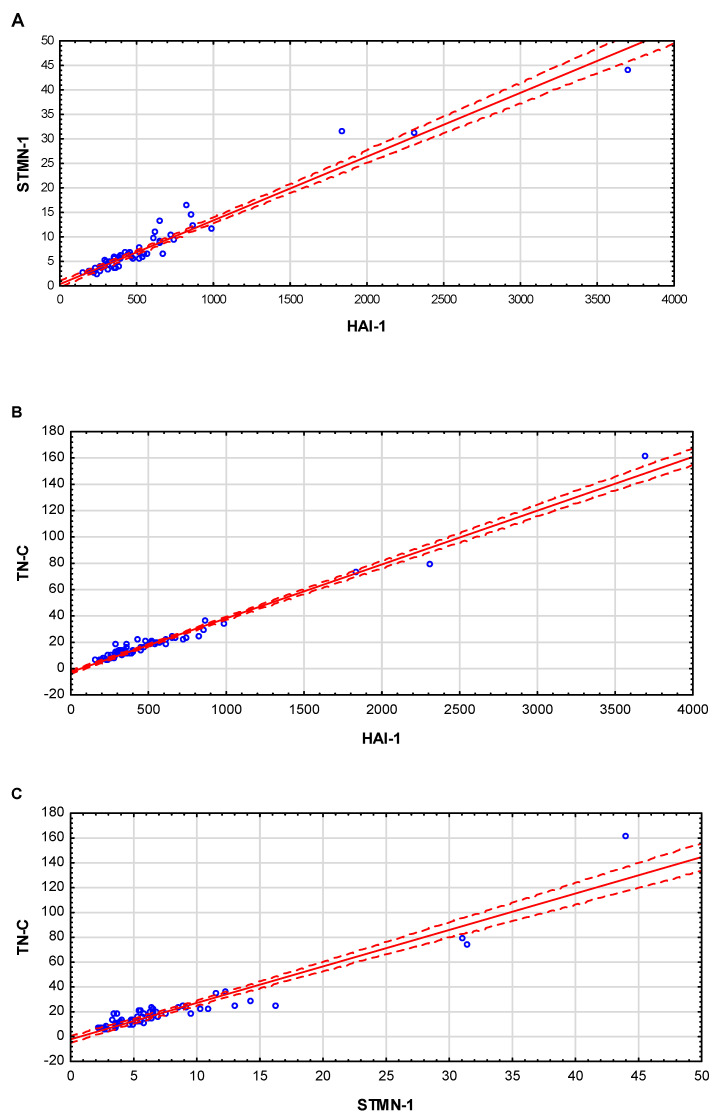
Correlations between parameters. (**A**)—linear relationship between concentration HAI-1 and STMN-1; (**B**)—linear relationship between HAI-1 and TN-C; (**C**)—linear relationship between STMN-1 and TN-C; the correlations were evaluated with Spearman’s nonparametric test.

Mutual positive correlations between HAI-1 STMN-1 and TN-C in the BC patients were demonstrated in Figure 2.

A full correlation was demonstrated (full correlation: 0.9 ≤ R ≤ 1) between HAI-1 and STMN-1, Spearman’s coefficient was R = 0.94 (*p* ≤ 0.001) (Figure 2A), and between HAI-1 and TN-C, R coefficient = 0.94 (*p* ≤ 0.001) (Figure 2B). A very high correlation (very high correlation 0.7 ≤ R ≤ 0.9) was demonstrated between STMN-1 and TN-C; Spearman’s coefficient was R = 0.89 (*p* ≤ 0.001) (Figure 2C).

The studies did not observe any correlation between variables such as age, smoking or sex with the studied parameters (*p* > 0.05).

### 3.4. Diagnostic Value of HAI-1, STM-1 and TN-C in BC and Subgroups NMIBC, MIBC, LG and HG

ROC analysis with AUC calculation was performed to investigate the ability of each parameter to discriminate BC patients and BC subgroups from healthy subjects (the results are summarised in Table 6). Figure 2 graphically presents the calculation of the diagnostic value of the parameters in the differential diagnosis.

**Figure 3:**
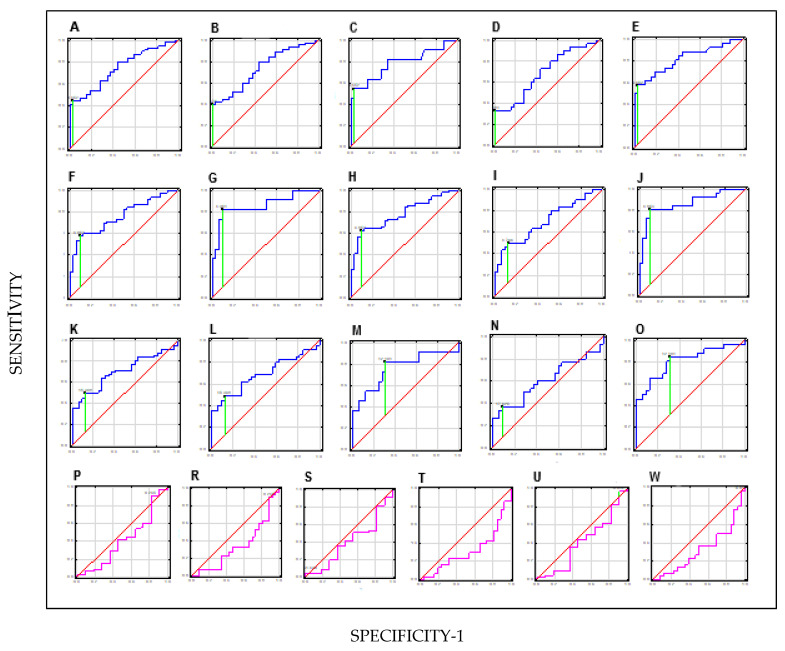
Receiver operating characteristic curves evaluating ability of HAI-1, STMN-1 and TN-C to distinguish BC patients from healthy people (C) (navy line, ROC), and NMIBC patients from MIBC and LG patients from HG (pink line, ROC); cut-of (green line).

The order of the ROC graphs corresponds to the order of the parameters presented in Table 6 (Figure 3).

The first five ROC graphs (first row) show the analysis of HAI-1 of the BC group and subgroups with different degrees of invasiveness and malignancy from the control [BC (A), NMIBC (B), MIBC (C), LG (D), HG (E)]. The graphs in the second row (6–10) show the analysis of STMN-1 of the BC group and subgroups with different degrees of invasiveness and malignancy from the control [STMN-1: BC (F), NMIBC (G), MIBC (H), LG (I), HG (J)]. In the third row, the next five graphs (11–15) show the analysis of TN-C of the BC group and subgroups with different degrees of invasiveness and malignancy from the control [TN-C: BC (K), NMIBC (L), MIBC (M), LG (N), HG (O)]. The last, 4th row shows the comparison of HAI-1, STMN-1 and TN-C parameters between subgroups with varying degrees of invasiveness and malignancy [HAI-1: NMIBCvsMIBC (P), LGvsHG (R); STMN-1: NMIBCvsMIBC (S), LGvsHG (T); TN-C: NMIB-CvsMIBC (U), LGvsHG (W)].

For HAI-1 determined in the urine of BC patients, the sensitivity was 47%, and the specificity was 98%. The proposed cut-off point was 0.452 ng/mg creatinine. The AUC for HAI-1 was 0.75 [95% confidence interval (CI) (0.65–0.85), *p* < 0.001]. STMN-1 determined in the urine of patients with BC had a sensitivity of 65% and specificity of 91%. The proposed cut-off point was 4.85 ng/mg creatinine. The AUC for STMN-1 was 0.78 [95% CI (0.68–0.87), *p* ≤ 0.001]. TN-C measured in the urine of patients with BC obtained a sensitivity of 51% and specificity of 88%. The proposed cut-off point was 15.368 ng/mg creatinine. The AUC for TN-C was 0.70 [95% CI (0.59–0.81), *p* ≤ 0.001]. For most diagnostic tests considered good, the AUC size range is <0.8–0.95>. For the tested individual parameters, the AUC was 0.75, 0.78 and 0.7, respectively, which indicates limited clinical usefulness (<0.8).

The good diagnostic values as parameters for distinguishing BC patients from healthy people (C) were determined to be (i) HAI-1 in the HG subgroup of patients with AUC = 0.82, 60% sensitivity, and 82% specificity, (ii) STMN-1 in the MIBC and HG subgroups of patients with AUC = 0.86, 85% sensitivity, 89% specificity and with AUC = 0.87, 81% sensitivity, 89% specificity, respectively, and (iii) TN-C in the HG subgroup of patients with AUC = 0.81, 87% sensitivity, and 69% specificity. AUC values > 0.8 are considered clinically useful.

However, the diagnostic value of the studied parameters in differentiating the BC subgroups NMIBC from MIBC and LG from HG was not demonstrated.

### 3.5. Diagnostic Value of Combinations of Parameters in BC and Subgroups

**Figure 4:**
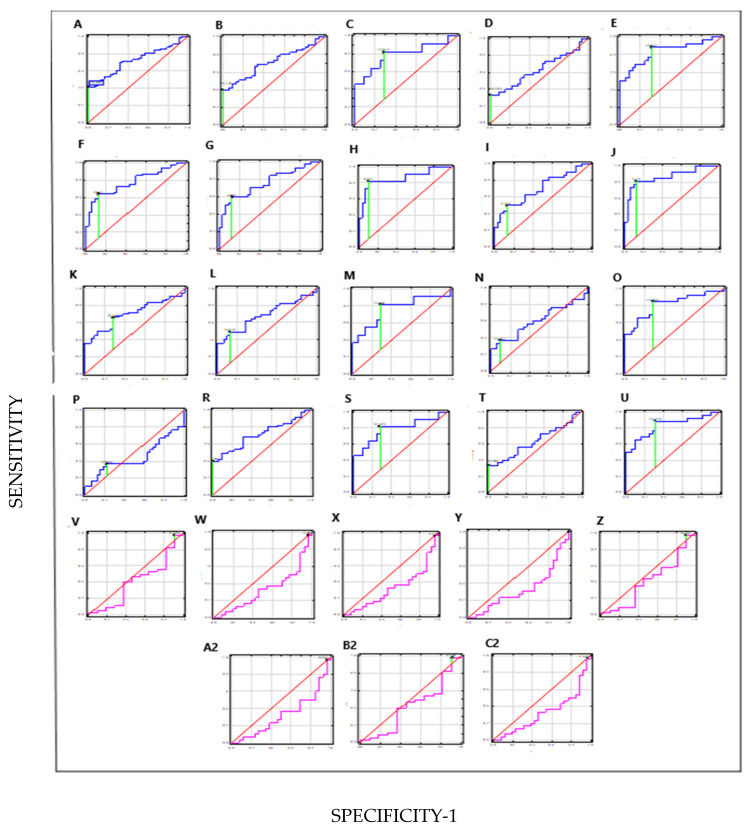
Receiver operating characteristic curves evaluating ability of combinations of parameters (HAI-1, STMN-1 and TN-C) to distinguish BC patients from healthy people (C) (navy line), and NMIBC patients from MIBC and LG patients from HG (pink line); cut-of (green line).

Modelling the combinations of parameters was the next step in the search for a diagnostic tool for differential diagnosis of BC and the defined BC subgroups compared to healthy people (C) and the distinctions between BC subgroups. The results are collected in Table 7. Figure 4 uses ROC curves to graphically visualise these models.

The order of the ROC graphs corresponds to the order of the parameter combinations presented in Table 7 (Figure 4).

[HAI-1+STMN-1+TN-C (A), HAI-1(NMIBC)+STMN-1(NIBC)+TN-C(NMIBC) (B), HAI-1(MIBC)+STMN-1(MIBC)+TN-C(MIBC) (C), HAI-1(LG)+STMN-1(LG)+TN-C(LG) (D), HAI-1(HG)+STMN-1(HG)+TN-C(HG) (E), HAI-1+STMN-1 (F), HAI-1(NMIBC)+STMN-1(NMIBC) (G), HAI-1(MIBC)+STMN-1(MIBC) (H), HAI-1(LG)+STMN-1(LG) (I), HAI-1(HG)+STMN-1(HG) (J), HAI-1+TN-C (K), HAI-1(NMIBC)+TN-C(NMIBC) (L), HAI-1(MIBC)+TN-C(MIBC) (M), HAI-1(LG)+TN-C(LG) (N), HAI-1(HG)+TN-C(HG) (O), STMN-1+TN-C (P), STMN-1(NMIBC)+TN-C(NMIBC) (R), STMN-1(MIBC)+TN-C(MIBC) (S), STMN-1(LG)+TN-C(LG) (T), STMN-1(HG)+TN-C(HG) (U), HAI-1(NMIBC)+STMN-1(NMIBC)+TN-C(NMIBC) vs. HAI-1(MIBC)+STMN-1(MIBC)+TN-C(MIBC) (V), HAI-1(LG)+STMN-1(LG)+TN-C(LG) vs. HAI-1(HG)+STMN-1(HG)+TN-C(HG) (W), HAI-1(NMIBC)+STMN-1(NMIBC) vs. HAI-1(MIBC)+STMN-1(MIBC) (X), HAI-1(LG)+STMN-1(LG) vs. HAI-1(HG)+STMN-1(HG) (Y), HAI-1(NMIBC)+TN-C(NMIBC) vs. HAI-1(MIBC)+TN-C(MIBC) (Z), HAI-1(LG)+TN-C(LG) vs. HAI-1(HG)+TN-C(HG) (A2), STMN-1(NMIBC)+TN-C(NMIBC) vs. STMN-1(MIBC)+TN-C(MBC) (B2), STMN-1(LG)+TM-C(HG) vs. STMN-1(HG)+TN-C(HG) (C2)].

The combination of HAI-1+STMN-1 in the HG subgroup of BC patients showed a clinically useful value in distinguishing BC from healthy individuals, AUC = 0.87, 89% sensitivity, and 85% specificity. Similarly, the combination of HAI-1+STMN-1 in the MIBC subgroup of BC patients had good diagnostic value, AUC = 0.86, 82% sensitivity, and 91% specificity. The combination of three parameters (HAI-1+STMN-1+TN-C) in the HG subgroup of BC patients also distinguished patients from healthy individuals well, with AUC = 0.83, 88% sensitivity, and 69% specificity. However, the combination of parameters did not enable us to distinguish the subgroups between NMIBC and MIBC and LG and HG and the AUC values, sensitivity and specificity were poor.

## 4. Discussion

Approx. 50–70% of BCs recur despite conservative treatment such as transurethral and intravesical therapy [41,42]. Unfortunately, for this reason, a patient diagnosed with BC must be monitored and followed throughout their life, which generates one of the highest pharmacoeconomic costs from diagnosis to death among all cancers. Therefore, there is a need to implement a diagnostic test to reduce the costs related to monitoring and improve the quality of life of patients by potentially minimising the number of invasive endoscopic assessments [8]. In addition, screening for people at high risk of BC (people with a history of tobacco abuse and high-risk occupational exposure) may be beneficial for the early detection of BC. The urine comes into direct contact with the bladder tumours, therefore urinary markers are of great interest in this field [9].

Urinary biomarkers can take many forms, such as proteins, metabolites, DNA, different types of RNA, and single nucleotide polymorphisms (SNPs). The presence of or changes in the expression of these molecules may be associated with BC [10]. To date, the only urinary markers that have Food and Drug Administration (FDA) approval for the diagnosis and monitoring of bladder cancer are BTAstat^®^, BTA TRAK^®^, NMP22 and UroVysion™, with ImmunoCyt™/uCyt+™ only approved for bladder cancer observation, including cytology, which remains the gold standard [9].

A group of six proteins (nuclear matrix proteins, BLCA) specific for BC has also become a promising molecular marker, with particular emphasis on BLCA-4 (Bladder Cancer Associated Protein 4), which is expressed at a very early stage of the disease and can be detected in urine before tumour formation [43].

The use of a combination of markers allows for a significant increase in the accuracy of detecting urinary abnormalities in patients. Investigated the usefulness of the nuclear matrix protein (NMP22) as a diagnostic marker in BC in terms of genetic susceptibility (slow acetylator N-acetyltransferase 2, NAT2) combined with detoxification abilities (Glutathione S-transferases, GST and the GST isoenzyme—π) [44]. They reported more than twice the level of NMP22 in the urine of patients with BC compared to the control group, and most of the patients were also slow acetylators. Another study demonstrating a higher efficiency in detecting BC using a panel of molecular markers that would reflect environmental risk showed a higher diagnostic value of nuclear matrix proteins in combination with the DNA damage marker (8-hydroxy-2′-deoxyguanosine, 8-OHdG) than the single proteins NMP22 and BLCA-4 [45].

Given the wide range of knowledge, including medical knowledge, artificial intelligence (AI) is becoming a promising source of information, and the latest evidence indicates its great potential in the diagnosis, prognosis and treatment of urological diseases, including BC [46].

A single marker turns out to be insufficient for the proper early diagnostic evaluation or monitoring of the course of BC, therefore finding a few parameters that make up a bladder cancer diagnostic panel seems to be the best solution. The HAI-1, STMN-1 and TN-C proteins, different in terms of their function, whose activity has been observed in various types of neoplasms, inspired us to study them in BC [14,17,19,23].

In our own study, the values of these selected proteins in the urine of bladder cancer patients were compared to urologically healthy controls. The influence of the invasiveness and malignancy of BC on the levels of the selected parameters was also assessed. In the search for a diagnostic tool for the differential diagnosis of BC, we also investigated the combination of the studied parameters.

In our own study, it was shown that the concentrations of these parameters in the urine of patients with BC were statistically significantly higher compared to the concentrations of the tested proteins in healthy subjects: HAI-1 (*p* ≤ 0.001), STMN-1 (*p* ≤ 0.001), and TN-C (*p* = 0.002). In the subgroups of patients with varying degrees of invasiveness and in subgroups with different grades, the HAI-1 and STMN-1 levels were also statistically significantly higher compared to the controls, but the HAI-1, STMN-1 did not differ significantly between the subgroups of NMIBC and MIBC, and the LG and HG patients. In the case of TN-C in the subgroup of LG patients, no statistically significant difference was found between the concentrations of this protein in the urine of patients compared to the control, but there was a statistically significant difference between the concentrations of TN-C in the subgroup of HG patients versus LG (*p* = 0.04). The concentration of TN-C was statistically significantly higher in the urine of the MIBC and NMIBC patients compared to the control, but no significant difference was found in relation to the values obtained in the healthy subjects. Based on the results, it can be concluded that the increases in HAI-1 and STMN-1 occur in the early stages of BC and increase with the progression of the neoplastic process. Both parameters could be potential diagnostic and prognostic markers in BC. On the other hand, TN-C could diagnose patients with a higher degree of malignancy.

Snell et al. [47] investigated whether HAI-1, EpCAM (Epithelial Cell Adhesion Molecule) and EGFR (epidermal growth factor receptor) can be independent prognostic biomarkers in patients with non-invasive bladder cancer (NMIBC) and whether they are used for risk stratification to help in making treatment decisions. Increased protein levels were also shown to be associated with an increased risk of death from NMIBC over 5 years in patients in the study. Moreover, it has been shown that increased HAI-1 and EGFR in the urine are also associated with increased mortality in patients with MIBC. In the case of HAI-1, such conclusions have not been reported before, however, they require further analysis for confirmation.

When studying HAI-1 expression, proteins that are activated by HAI-1 and the consequences of the cascade of induced reactions are of great importance. In immunohistochemical analyses of tumours from patients with invasive BC by Yamasaki et al. [48], the expression of phosphorylated-Met (phospho-Met) and HAI-1 matriptase was assessed, and the increased expression of matriptase was significantly associated with poor prognosis in patients with invasive BC. Immunohistochemical studies showed an enhanced expression of HAI-1 and phospho-Met, confirming the importance of HAI-1-induced regulation of Met phosphorylation as a major synergistic role in BC progression.

Moreover, it has been shown that the increased expression of matriptase can induce ligand-dependent Met activation. These findings suggest that HAI-1 may regulate ligand-dependent Met activation in MIBC patients [49].

The soluble Met concentration has also been described as a biomarker in the urine for the detection of MIBC [50,51].

Schimwell et al. [52] tested the combination of tumour gene expression profiling with proteomic analysis of tumour cell line secretions as a strategy to discover urinary biomarkers of BC. Secreted proteins with high mRNA levels in bladder tumours compared with normal urinary tract epithelium were determined by the ELISA method in urine samples from patients. HAI-1 was significantly increased in patients with bladder cancer, with the highest values in invasive disease.

On the other hand, in a study by Hemdan et al. [53], the immunohistochemical evaluation of STMN-1 expression in diseased tissue preparations from patients with BC was performed. Clearly, increased STMN-1 activity was observed in bladder cancer tissue, both in non-invasive and invasive tumours, as well as in BC metastases. High STMN-1 expression correlated with a shorter survival rate.

Dubosq et al. [54] report that STMN-1 is part of a trigene signature predicting early tumour relapse. Regarding prognostic markers, markers of relapse and progression are needed in non-invasive tumours, while in the case of muscle-infiltrating cancer, the emphasis is on factors that identify the risk of metastasis and death.

Brunner et al. [55] studied the expression of TN-C by immunohistochemistry in BC patients using an anti-TN-C monoclonal antibody and estimated the overall and relapse-free survival. The analysis showed that invasive tumours with diffuse TN-C staining the stroma had a much worse prognosis than those that were negative or poorly pigmented.

In our own work, strong positive correlations between the examined parameters were shown, which may indicate the parallel participation of such functionally different proteins in the course of bladder cancer.

The priority of our study was to assess the diagnostic utility of HAI-1, STMN-1 and TN-C. We assessed both single parameters as well as their combinations in the differential diagnosis of BC. For most diagnostic tests considered good, the AUC size range is <0.8–0.95>. For the single parameters (HAI-1, STMN-1 and TN-C) in the group of BC patients in relation to the healthy control, the AUCs were 0.75, 0.78 and 0.7, respectively, which indicates limited clinical usefulness (<0.8). However, better diagnostic utility was demonstrated by HAI-1 in the HG subgroup of patients with AUC = 0.82, STMN-1 in the MIBC and HG subgroups of patients with AUC = 0.86 and AUC = 0.87, respectively, and TN-C in the HG subgroup of patients with AUC = 0.81. Of the three parameters studied, urinary STMN-1 showed the best diagnostic value for detecting BC.

In the combination of parameters, similar AUC values were obtained compared to the single parameters: HAI-1+STMN-1 (AUC = 0.78), HAI-1+STMN-1+TN-C (AUC = 0.73), HAI-1+TN-C (AUC = 0.70), while the combination of STMN-1+TN-C (AUC = 0.45) was the weakest. On the other hand, the combination of HAI-1+STMN-1 in the HG subgroup of BC patients showed a good diagnostic value in distinguishing them from healthy individuals, with AUC = 0.87, 89% sensitivity, and 85% specificity.

Although the selected parameters show potential value as indicators in BC detection, they cannot be used to distinguish the degree of invasiveness and malignancy of BC.

Patient age, sex, and cigarette smoking were excluded as potential confounding factors that could affect the values of the studied parameters, which increase their diagnostic value in detecting BC.

The presented studies have some limitations, such as a relatively small number of patients, and a lack of assessment of the impact of possible diseases such as diabetes, cardiovascular diseases, obesity or medication. The presented results are only an introduction to further studies and constitute a preliminary analysis of the usefulness of HAI-1, STMN-1 and TN-C as diagnostic and prognostic markers in BC.

## 5. Conclusions

The urine comes into direct contact with bladder tumours, therefore the measurement of parameters in urine seems to be extremely useful in this endeavour. Increased concentrations of HAI-1, STMN-1 and TN-C (especially STMN-1 and the combination of HAI-1+STMN-1) in the urine of patients with BC may indicate the appearance of neoplastic changes in the bladder, and thus provide additional prognostic information on the results of treatment and possible disease recurrence. A panel of these three selected proteins showed their correlation in the process of BC tumorigenesis, as evidenced by the strong relationships between them. However, in order to learn more about the contribution of these parameters to the progression of bladder cancer, it is appropriate to continue the research that has already been started on a larger number of patients with BC.

## Figures and Tables

**Table 1:** Characteristics of bladder cancer patients group (BC) and control group.

Population Characteristic	BC	C
N(%)	(100) 56	(100) 32
age range (median years)	41–88 (69)	50–81 (67)
smoking	43 (77)	26 (81)
non-smoking	13 (23)	10 (19)
Men	48 (86)	27 (84)
Women	8 (14)	5 (16)
Clinical grading		
LG	30 (54)	
HG	26 (46)	
Clinical subgroups		
NMIBC	45 (80)	
MIBC	11(20)	

N—number of patients; BC—bladder cancer; C—control group; LG—low grade; HG—high grade; NMIBC—non-muscle invasive bladder cancer; MIBC—muscle-invasive bladder cancer.

**Table 2:** Results of HAI-1 of BC patients, subgroups and control group with statistical analysis.

HAI-1 [ng/mg Creat.]	Mean ± SD	Median (IQR)	*p*
BC	0.56 ± 0.56	0.39 (0.29–0.61)	≤0.001 *
C	0.29 ± 0.88	0.28 (0.22–0.37)	
smoking patients of BC	0.57 ± 0.63	0.36 (0.27–0.62)	≤0.001 ** BC(S):C = 0.004 BC(nonS):C ≤ 0.001 BC(S):BC (nonS) = NS
non-smoking patients of BC	0.49 ± 0.16	0.45 (0.35–0.57)	
Women of BC	0.94 ± 0.12	0.40 (0.36–0.86)	≤0.001 ** BC(w):C = 0.002 BC(m):C = 0.001 BC(w):BC(m) = NS
Men of BC	0.50 ± 0.38	0.37 (0.29–0.61)	
NMIBC	0.50 ± 0.52	0.38 (0.29–0.55)	0.0004 ** NMIBC:C = 0.001 MIBC:C = 0.006 NMIBC:MIBC = NS
MIBC	0.65 ± 0.59	0.45 (0.33–0.82)	
LG	0.40 ± 0.17	0.36 (0.27–0.51)	≤0.001 ** LG:C = 0.046 HG:C ≤ 0.001 LG:HG = NS
HG	0.68 ± 0.73	0.46 (0.33–0.72)	

HAI-1—Human hepatocyte growth activator inhibitor 1; BC—bladder cancer; C—control group; BC(S)—smoking patients of BC; BC(nonS)—non-smoking patients of BC; BC(w)—women of BC; BC(m)—men of BC; NMIBC—non-muscle invasive bladder cancer; MIBC—muscle-invasive bladder cancer; LG—low grade; HG—high grade; IQR—interquartile ranges; NS—not statistically significant; *p*—statistically significant difference; *—U Mann–Whitney test; **—Kruskal-Wallis test.

**Table 3:** Results of STMN-1 of BC patients, subgroups and control group with statistical analysis.

STMN-1 [ng/mg Creat.]	Mean ± SD	Median (IQR)	*p*
BC	7.65 ± 4.45	5.52 (3.75–8.13)	≤0.001 *
C	3.60 ± 1.20	3.61 (2.76–4.15)	
smoking patients of BC	7.94 ± 8.41	5.35 (3.53–8.93)	≤0.001 * BC(S):C ≤ 0.001 BC(nonS):C ≤ 0.001 BC(S):BC (nonS) = NS
non-smoking patients of BC	6.68 ± 3.19	6.39 (4.85–6.54)	
Women of BC	11.37 ± 14.72	5.32 (3.59–12.29)	≤0.001 * BC(w):C = 0.013 BC(m):C ≤ 0.001 BC(w):BC(m) = NS
Men of BC	7.11 ± 6.00	5.52 (3.75–7.77)	
NMIBC	6.72 ± 6.38	5.42 (3.71–6.12)	≤0.001 ** NMIBC:C ≤ 0.001 MIBC:C = 0.001 NMIBC:MIBC = NS
MIBC	9.35 ± 8.23	5.83 (4.95–12.30)	
LG	5.47 ± 2.87	4.45 (3.59–6.40)	≤0.001 ** LG:C = 0.024 HG:C ≤ 0.001 LG:HG = NS
HG	9.27 ± 9.14	6.43 (4.95–9.21)	

STMN-1—Stathmin 1; BC—bladder cancer; C—control group; BC(S)—smoking patients of BC; BC(nonS)—non-smoking patients of BC; BC(w) –women of BC; BC(m)—men of BC; NMIBC—non-muscle invasive bladder cancer; MIBC—muscle-invasive bladder cancer; LG—low grade; HG—high grade; IQR—interquartile ranges; NS—not statistically significant; *p*—statistically significant difference; *—U Mann–Whitney test; **—Kruskal-Wallis test.

**Table 4:** Results of TN-C of BC patients, subgroups and control group with statistical analysis.

TNC [ng/mg Creat.]	Mean ± SD	Median (IQR)	*p*
BC	20.29 ± 23.24	14.66 (10.85–21.62)	0.002 *
C	11.25 ± 3.71	11.04 (8.39–14.32)	
smoking patients of BC	21.49 ± 26.33	15.37 (9.35–21.96)	0.005 ** BC(S):C = 0.022 BC(nonS):C = 0.017 BC(S):BC (nonS) = NS
non-smoking patients of BC	16.34 ± 5.17	13.96 (12.10–19.55)	
Women of BC	38.39 ± 54.53	18.14 (11.40–36.29)	0.004 ** BC(w):C = 0.028 BC(m):C = 0.015 BC(w):BC(m) = NS
Men of BC	17.71 ± 14.02	13.82 (9.68–21.620)	
NMIBC	18.43 ± 22.6	13.68 (10.85–20.08)	0.005 ** NMIBC:C = 0.018 MIBC:C = 0.022 NMIBC:MIBC = NS
MIBC	22.99 ± 20.41	16.05 (12.10–24.56)	
LG	14.03 ± 6.69	12.52 (9.35–18.96)	≤0.001 ** LG:C = NS HG:C ≤ 0.001 LG:HG = NS
HG	25.44 ± 30.80	18.04 (12.19–23.30)	

TN-C—Tenascin C; BC—bladder cancer; C—control group; BC(S)—smoking patients of BC; BC(nonS)—non-smoking patients of BC; BC(w) –women of BC; BC(m)—men of BC; NMIBC—non-muscle invasive bladder cancer; MIBC—muscle-invasive bladder cancer; LG—low grade; HG—high grade; IQR—interquartile ranges; NS—not statistically significant; *p*—statistically significant difference; *—U Mann–Whitney test; **—Kruskal-Wallis test.

**Table 5:** Correlations between parameters.

Parameters	R	*p*	
HAI-1 (MIBC) vs. STMN-1 (MIBC)	0.972	≤0.001	almost complete dependence
HAI-1 (MIBC) vs. TN-C (MIBC)	0.956	≤0.001	
STMN-1 (MIBC) vs. TN-C (MIBC)	0.955	≤0.001	
HAI-1 (HG) vs. STMN-1 (HG)	0.943	≤0.001	
HAI-1 vs. TN-C	0.942	≤0.001	
HAI-1 vs. STMN-1	0.941	≤0.001	
HAI-1 (LG) vs. TN-C(LG)	0.938	≤0.001	
HAI-1 (NMIBC) vs. TN-C (NMIBC)	0.930	≤0.001	
HAI-1 (HG) vs. TN-C (HG)	0.912	≤0.001	
HAI-1 (NMIBC) vs. STMN-1 (NMIBC)	0.911	≤0.001	
STMN-1 vs. TN-C	0.890	≤0.001	very high dependence
STMN-1(HG) vs. TN-C (HG)	0.887	≤0.001	
STMN-1 (NMIBC) vs. TN-c (NMIBC)	0.857	≤0.001	
HAI-l (LG) vs. STMN-1 (LG)	0.883	≤0.001	
STMN-1 (LG) vs. TN-C (LG)	0.842	≤0.001	

HAI-1—Human hepatocyte growth activator inhibitor 1; STMN-1—Stathmin 1; TN-C—Tenascin; R—Spearman coefficient.

**Table 6:** Diagnostic efficiency of HAI-1, STMN-1 and TN-C to distinguish BC patients from control and distinctions between BC subgroups.

	Sensitivity	Specificity	PPV	NPV	AUC	*p*
	HAI-1					
BC vs. C	47%	98%	96%	50%	0.75	0.0000
NMIBC vs. C	43%	98%	100%	54%	0.74	0.0000
MIBC vs. C	57%	97%	86%	86%	0.79	0.0021
LG vs. C	35%	99%	100%	62%	0.69	0.0043
HG vs. C	60%	82%	94%	74%	0.82	0.0000
NMIBC vs. MIBC	82%	9%	18%	33%	0.41	0.3879
LG vs. HG	88%	10%	54%	50%	0.34	0.3460
	STMN-1					
BC vs. C	65%	91%	92%	58%	0.78	0.0000
NMIBC vs. C	60%	91%	90%	60%	0.76	0.0000
MIBC vs. C	85%	89%	75%	94%	0.86	0.0000
LG vs. C	53%	88%	88%	60%	0.70	0.0023
HG vs. C	81%	89%	89%	85%	0.87	0.0000
NMIBC vs. MIBC	9%	96%	10%	20%	0.40	0.3177
LG vs. HG	0%	100%	-	-	0.32	0.0123
	TN-C					
BC vs. C	51%	88%	88%	14%	0.70	0.0020
NMIBC vs. C	51%	88%	85%	55%	0.68	0.0018
MIBC vs. C	85%	69%	47%	92%	0.78	0.0079
LG vs. C	38%	88%	24%	60%	0.61	0.1516
HG vs. C	87%	69%	69%	85%	0.81	0.0000
NMIBC vs. MIBC	98%	9%	81%	50%	0.41	0.3902
LG vs. HG	96%	3%	54%	50%	0.33	0.0157

HAI-1—Human hepatocyte growth activator inhibitor 1; STMN-1—Stathmin 1; TN-C—Tenascin; BC—bladder cancer; C—control group; NMIBC—non-muscle invasive bladder cancer; MIBC—muscle-invasive bladder cancer; LG—low grade; HG—high grade; PPV—positive predictive value; NPV—negative predictive value; AUC—area under the curve.

**Table 7:** Diagnostic efficiency combinations parameters (HAI-1, STMN-1 and TN-C) to distinguish BC patients from control (C) and distinctions between BC subgroups.

Combinations Parameters	Sensitivity	Specificity	PPV	NPV	AUC	*p*
HAI-1+STMN-1+TN-C vs. C	41%	99%	100%	49%	0.73	0.0000
HAI-1(NMIBC)+STMN-1(NMIBC)+TN-C(NMIBC) vs. C	40%	99%	100%	54%	0.71	0.0002
HAI-1(MIBC)+STMN-1(MIBC)+TN- C(MIBC) vs. C	82%	72%	50%	92%	0.78	0.0020
HAI-1(LG)+STMN-1(LG)+TN-C(LG) vs. C	33%	99%	100%	62%	0.64	0.0603
HAI-1(HG)+STMN-1(HG)+TN-C(HG) vs. C	88%	69%	70%	88%	0.83	0.0000
HAI-1+STMN-1 vs. C	64%	87%	90%	58%	0.78	0.0000
HAI-1(NMIBC)+STMN-1(NIMBC) vs. C	61%	88%	87%	61%	0.76	0.0000
HAI-1(MIBC)+STMN-1(MIBC) vs. C	82%	91%	75%	94%	0.86	0.0000
HAI-1(LG)+STMN-1(LG) vs. C	50%	87%	79%	65%	0.71	0.0021
HAI-1(HG)+STMN-1(HG) vs. C	89%	85%	84%	85%	0.87	0.0000
HAI-1+TN-C vs. C	66%	72%	80%	55%	0.70	0.0002
HAI-1(NMIBC)+STMN-1(NMIBC) vs. C	49%	88%	92%	55%	0.69	0.0072
HAI-1(MIBC)+TN-C(MIBC) vs. C	82%	72%	43%	91%	0.71	0.0072
HAI-1(LG)+TN-C(LG) vs. C	37%	91%	38%	88%	0.61	0.1513
HAI-1(HG)+TN-C(HG) vs. C	85%	72%	71%	85%	0.81	0.0000
STMN-1+TN-C vs. C	37%	78%	100%	49%	0.45	0.4883
STMN-1(NMIBC)+TN-C(NMIBC) vs. C	43%	98%	100%	53%	0.71	0.0003
STMN-1(MIBC)+TN-C(MIBC) vs. C	82%	72%	50%	92%	0.78	0.0020
STMN-1(LG)+TN-C(LG) vs. C	33%	99%	100%	64%	0.63	0.0652
STMN-1(HG)+TN-C(HG) vs. C	88%	70%	70%	88%	0.83	0.0000
HAI-1(NMIBC)+STMN-1(NMIBC)+TN-C(NMIBC) vs. HAI-1(MIBC)+STMN-1(MIBC)+TN-C(MIBC)	98%	10%	81%	50%	0.41	0.3725
HAI-1(LG)+STMN-1(LG)+TN-C(LG) vs. HAI-1(HG)+STMN-1(HG)+TN-C(HG)	97%	4%	54%	50%	0.32	0.0152
HAI-1(NMIBC)+STMN-1(NMIBC) vs. HAI-1(MIBC)+STMN-1(MIBC)	84%	19%	80%	22%	0.41	0.3375
HAI-1(LG)+STMN-1(LG) vs. HAI-1(HG)+STMN-1(HG)	100%	0%	-	-	0.32	0.0156
HAI-1(NMIBC)+TN-C(NMIBC) vs. HAI-1(MIBC)+TN-C(MIBC)	97%	9%	81%	33%	0.42	0.3721
HAI-1(LG)+TN-C(LG) vs. HAI-1(HG)+TN-C(HG)	97%	4%	54%	50%	0.33	0.0157
STMN-1(NMIBC)+TN-C(NMIBC) vs. STMN1(MIBC)+TN-C(MIBC)	98%	10%	81%	50%	0.41	0.3725
STMN-1(LG)+TN-C(LG) vs. STMN-1(HG)+TN-C(HG)	97%	4%	54%	50%	0.32	0.0152

HAI-1—Human hepatocyte growth activator inhibitor 1; STMN-1—Stathmin 1; TN-C—Tenascin; BC—bladder cancer; C—control group; NMIBC—non-muscle invasive bladder cancer; MIBC—muscle-invasive bladder cancer; LG—low grade; HG—high grade; PPV—positive predictive value; NPV—negative predictive value; AUC—area under the curve.

## Data Availability

The datasets generated during and/or analysed during the current study are available from the corresponding author upon reasonable request.
